# A Literature Review of Online Identity Reconstruction

**DOI:** 10.3389/fpsyg.2021.696552

**Published:** 2021-08-23

**Authors:** Jiao Huang, Sameer Kumar, Chuan Hu

**Affiliations:** ^1^School of Information Management, Jiangxi University of Finance and Economics, Nanchang, China; ^2^Asia-Europe Institute, University of Malaya, Kuala Lumpur, Malaysia; ^3^School of Management, Nanchang University, Nanchang, China

**Keywords:** identity reconstruction, false self-presentation, strategic self-presentation, internet, literature review

## Abstract

The tremendous development of the Internet enables people to present themselves freely. Some people may reconstruct their identity on the Internet to build an online identity that is partly or even completely different from their real identity in the offline world. Given that research on online identity reconstruction is fragmented, it is important to evaluate the current state of the literature. In this paper, a review of literature related to online identity reconstruction was conducted. This study summarized the theoretical and methodological preferences of relevant research. In addition, it elaborated why and how people engage in online identity reconstruction. The predictors and effects of online identity reconstruction were also discussed. The results of this study provided an overview of the thematic patterns of existing research. This review also identified current research gaps and recommended possible directions for future studies.

## Introduction

Online identity refers to “a configuration of the defining characteristics of a person in the online space” (Kim et al., 2011). Ruyter and Conroy (2002) defined online identity as the combination of characteristics that help to define a person in cyberspace, thereby, makes him or her different from other online users. The rapid development of information technology has provided people with various tools to create their online identity and present themselves.

It is suggested that an individual’s identity in the online world may be different from his or her offline identity (Kim et al., 2011; Hu et al., 2015). An individual’s offline identity is constrained by his or her corporal body and the physical situations (Bargh et al., 2002; Donath, 2002; Schau and Gilly, 2003; Kim et al., 2011). Factors (e.g., race, age, and gender) that affect an individual’s offline identity are usually beyond his or her control (Kim et al., 2011). However, in the online world, people can construct and present their online identity selectively (Kim et al., 2011; Hu et al., 2015). In this case, the virtual identity that an individual builds online is not necessarily tied to his or her offline identity (Hongladarom, 2011). An individual can use different digital means to create an identity that he or she wishes to express online (Kim et al., 2011).

The phenomenon that people build an online identity that is partly or even completely different from their real identity by hiding or faking certain characteristics is defined as online identity reconstruction (Hu et al., 2015). It should be noted that an individual’s identity is “fluid” rather than “static.” It is socially constructed in a given context (Hatoss, 2012). Individuals often present themselves differently in different situations. Online identity reconstruction is different from online identity construction. Identity construction is a complex process in which people develop self-definition (Slay and Smith, 2011). It is usually related to personal attributes and social roles (Simpson and Carroll, 2008). For example, a teacher constructs his/her identity as a teacher researcher by participating in teacher education programs (Taylor, 2017); software engineering students build up their professional identity through training (Tomer and Mishra, 2016).

The studies about identity construction online mainly focus on how individuals build their self-image online. For example, adolescents create their online identity with the disclosure of intimate information and the use of various web-based resources (Alvermann et al., 2012; Jordán-Conde et al., 2014). People use different profile photos to present themselves (Hum et al., 2011), utilize various photographic and textual material to construct an alcohol-identity (Ridout et al., 2012), and edit the messages carefully in online interactions (Ditchfield, 2020).

Prior studies about online identity reconstruction paid more emphasis on the factors that make people’s online and offline identities different, such as strategic self-presentation (Kim and Baek, 2014; Fox and Rooney, 2015; Bareket-Bojmel et al., 2016), deceptive self-presentation (Toma and Hancock, 2010; Ranzini and Lutz, 2017), or false self-presentation (Gil-Or et al., 2015; Jackson and Luchner, 2018; Wright et al., 2018). For example, an unattractive girl may reconstruct her identity online by posting edited photos that make her look more attractive. Some people also regard online identity reconstruction as a way to explore their identity (Valkenburg et al., 2005; Valkenburg and Peter, 2008). For instance, adolescents may pretend to be someone else online to try different aspects of their possible identity (Valkenburg and Peter, 2008). Existing research has examined the associations between online identity reconstruction and various factors, such as well-being (Kim and Lee, 2011; Jang et al., 2018), sense of identity (Valkenburg and Peter, 2008), self-esteem (Kim and Baek, 2014; Gil-Or et al., 2015), and so on.

Although, increasingly more attention has been paid to online identity reconstruction, it is difficult for readers (researchers and practitioners) to understand the phenomenon of online identity reconstruction thoroughly, given that the existing research is quite diverse and fragmented. An extensive review of research in this area is thus necessary. A review of the growing literature can provide an overview of the current status of research concerning online identity reconstruction (such as methodological preference and research themes) and help researchers recognize research trends in this area. Therefore, this study aims to review what is already known about the phenomenon of online identity reconstruction. Specifically, the current study attempts to analyze the motivations, strategies, predictors, and effects of online identity reconstruction. Moreover, it will identify research gaps in the existing literature and provide recommendations for future research at the same time.

## Online Identity Reconstruction

On the Internet, physical cues are absent (Suler, 2004). People cannot physically see or hear each other which increases the perceived distance between people and the audiences (Bullingham and Vasconcelos, 2013). In addition, people’s online identity is usually determined by the information they disclose to others (Marwick, 2013). The physical detachment from audiences and the control on self-presentation makes it easier to hide or fake personal characteristics.

Previous research suggested that game characters created by players are more similar to their ideal self than to their actual self (Bessière et al., 2007). Moreover, research concerning online dating sites indicated that some people may engage in deceptive self-presentation, providing exaggerated or false information about themselves (Yurchisin et al., 2005; Toma et al., 2008). For example, people tend to present the “hoped-for possible self” on dating websites (Yurchisin et al., 2005). Some people lie about their height, while some lie about their weight to make themselves more attractive (Toma et al., 2008).

In comparison with other online self-presentation tools, social network platforms enable people to present themselves in a more structured and personalized way (Manago et al., 2008). In online profiles, people can share their basic personal information (such as gender, age, education, etc.), describe their preferences (e.g., people they are interested in), list their own interests (such as hobbies and favorite movies), and so on. In addition to profiles, people can also present themselves through other features on social network platforms, such as updating their status and sharing photos. Given that users can decide what information to disclose, the construction of identity on social network platforms is flexible (Manago et al., 2008). This means that identity reconstruction becomes possible because people can design and create their own online identity. People can hide or even alter their identity if they want (Suler, 2004). Previous research suggested that, on Facebook, individuals tended to build an online identity that is more socially desirable than their offline identity to make themselves appear more popular (Zhao et al., 2008). Given that individuals are able to reconstruct their online identity based on their own ideas, their identity on social network platforms could be partly, or even completely, different from their existing identity in the offline world (Hu et al., 2015).

### Online Identity Reconstruction and Strategic Self-Presentation

Before the concept of online identity reconstruction was proposed, the terms “strategic self-presentation” and “selective self-presentation” were frequently used in studies about building a different identity online. As suggested by Goffman (1959), people are concerned about their public images. To control the impressions they make on others, individuals tend to employ various strategies for self-presentation during online interactions, such as emphasizing the attractive aspects of themselves (Rui and Stefanone, 2013). Individuals who are not satisfied with certain characteristics of themselves are more likely to engage in self-enhancement online (Bessière et al., 2007). For instance, on social network platforms, people selectively post favorable personal information in their profiles and share positive life events more than negative ones (Bareket-Bojmel et al., 2016).

In addition to strategic self-presentation, the online environment also provides opportunities for online identity experiment, which refers to the tendency to pretend to be someone else in the online world. In comparison to offline contexts, online contexts are less limited by time and geographical distance, creating more opportunities for identity exploration (Shapiro and Margolin, 2014). In online contexts, people perceive increased disconnectedness from offline contexts and lower levels of surveillance (Selwyn, 2008). Therefore, young people tend to feel that there is less adult supervision online, which enables them to experiment with new values, ideas, and behaviors to a greater extent (Valkenburg and Peter, 2008). For example, a previous study showed that sexual minority adolescents (such as homosexuals) felt more comfortable expressing their sexuality to others in online contexts (Hillier and Harrison, 2007).

When engaging in strategic self-presentation or identity experiment, individuals intentionally build an online identity that is different from their offline identity to some extent. Therefore, they are actually engaging in online identity reconstruction. However, online identity reconstruction is more complex than strategic self-presentation and online identity experiment.

When presenting themselves strategically, people are still telling the truth, but mainly highlighting positive facts or exaggerating the truth on purpose. However, in online identity reconstruction, people are no longer limited to the truth. They may stretch the truth, hide personal information, or even tell lies. In addition, the motivations for online identity reconstruction are more complicated. The primary motivation for strategic self-presentation is to build a positive public image. For online identity experiment, individuals mainly want to talk and behave freely to explore the reactions of others (Valkenburg et al., 2005; Ceyhan, 2014). When engaging in online identity reconstruction, people are seeking the benefits brought about by the reconstructed identity, which is more than a positive image or the freedom to talk and behave. Some people try to pursue positive outcomes of online identity reconstruction, such as the fulfillment of vanity needs and access to new social networks (Hu et al., 2015). Some people reconstruct their identity to avoid negative outcomes, such as privacy risks (Hu et al., 2015).

## Methodology

To capture an overview of the diverse research concerning online identity reconstruction, this study aims to address five research questions as listed below. However, it should be mentioned that this study only answers the research questions based on the findings of the literature surveyed. More empirical research is needed to obtain definitive answers to these questions.

RQ1: What research methods and theories have been used in online identity reconstruction research?
RQ2: Why do people reconstruct their identity online?
RQ3: How do people reconstruct their identity online?
RQ4: What factors will affect online identity reconstruction behavior?
RQ5: What are the effects of online identity reconstruction?

### Search Strategy and Keywords

To collect relevant sources about online identity reconstruction, several academic databases were selected in this study, including Web of Science, Scopus, Academic Search Complete, and ScienceDirect. These four databases were selected because they cover a wide range of disciplines and a large number of academic journals. For example, Web of Science includes over 20,000 peer-reviewed journals in over 250 disciplines, while Scopus is a comprehensive abstract and citation database that covers more than 23,000 peer-reviewed journals. The use of multiple databases reduces the risk of omitting research related to online identity reconstruction.

The focus of the current study is on the issues related to creating or using an online identity that is somehow different from people’s offline identity. Some existing studies have investigated how people establish a general online identity through different technologies, such as profile photos (Hum et al., 2011), status updates (Yuan, 2018), and blogs (Fullwood et al., 2009; Sima and Pugsley, 2010). However, the construction or creation of a general online identity is not the focus of the present study. Therefore, the term “identity reconstruction/re-creation,” rather than “identity construction/creation,” was combined with terms that suggest an online context (such as “online” and “Internet”). Various terms were used before the concept of online identity reconstruction was proposed, such as “strategic self-presentation,” “deceptive self-presentation,” “false self-presentation,” and “identity experiment.” People may employ different strategies to build a different identity in the online context. When engaging in strategic self-presentation, individuals usually present positive aspects of the self to manage the impression they make on others (Bareket-Bojmel et al., 2016). In deceptive and false self-presentation, people provide inaccurate information about themselves, such as lying about their age, height, weight, occupation, and achievements (Toma and Hancock, 2010; Wright et al., 2018). Identity experiments are primarily used by adolescents. They explore and experiment with different identities on the Internet by emphasizing, changing, or concealing certain features of the self (Valkenburg and Peter, 2008; Ceyhan, 2014). Therefore, the terms that imply deviations between the online and offline identities were also included as keywords to search titles and abstracts in the selected databases.

### Inclusion and Exclusion Criteria

To ensure the quality of the sources for review, the search results were limited to research papers published in peer-reviewed journals. Therefore, to be included in the review, the article must be: (a) published in a peer-reviewed academic journal by 31st December 2020; (b) written in English; and (c) mainly focusing on the investigation of the reconstructed online identity that is partly or even totally different from people’s offline identity. Exclusion criteria ensured that selected articles are not: (a) book chapters, conference papers, review articles, dissertations, or non-academic studies; (b) focusing on identity reconstruction in the offline world; and (c) only focusing on the general strategies for self-presentation that are not intended to build a different online identity.

### Study Selection and Data Extraction

The literature search process in the selected databases identified 299 studies. After removing duplicates, 145 articles were left. The titles, abstracts, and keywords of the articles were screened, resulting in the exclusion of 69 sources that are clearly irrelevant. After that, the authors examined the full text of the remaining 76 papers independently to determine their relevance based on the eligibility criteria. Disagreements were resolved through discussion. In this process, 39 articles were excluded. Most of the excluded articles were focusing on the general strategies for online self-presentation, rather than online identity reconstruction. For example, some studies investigated how people present themselves to multiple audiences; some research examined the strategies for establishing a general online identity; some examined gender differences in self-presentation strategies between male and female users.

Finally, 37 papers were selected for review. The papers were examined thoroughly. The following variables were coded for each study: author(s), year of publication, research context, participant details, methodology, theoretical background, and key findings.

## Results

### Demographic

About the research context, the most frequently used research site is a social network platform. Sixteen studies (43.3%) were conducted on a specific social network platform (such as Facebook, Instagram, MySpace, WeChat, and QQ), while six studies (16.2%) focused on the general context of social network platforms. Additionally, seven articles (18.9%) investigated online dating sites or apps; five papers (13.5%) have examined online identity reconstruction in the general online environment and three studies (8.1%) looked into online gaming contexts. Most of the studies that looked into online identity reconstruction in online dating sites or the general online environment were published before the year 2015. Twenty-one of the reviewed papers (56.8%) were published after 2015, and they mainly investigated online identity reconstruction in the context of social network platforms.

When it comes to the samples, over one-third of the reviewed articles (13 studies, 35.1%) used a student sample. Among the articles that used a general sample, eight studies (21.6%) recruited the participants through crowdsourcing services, such as Amazon.com‘s Mechanical Turk, Qualtrics. The sample sizes of reviewed articles ranged from 10 to 1,158, depending on the applied methodology (i.e., qualitative or quantitative).

### Theoretical and Methodological Preference

The quantitative approach was the major methodology applied in previous research on online identity reconstruction. A total of 28 studies (75.7%) used quantitative research methods for data collection and data analysis, while seven studies (18.9%) employed qualitative methods and only two studies (5.4%) used mixed-methods (both quantitative and qualitative) for investigation. The most frequently used method for data collection was survey, followed by experiment, interview, focus group, and observation.

Ten studies did not have a clear theoretical foundation to explain the phenomenon of online identity reconstruction. The remaining studies mainly relied on theory of Goffman (1959) about self-presentation and different concepts of the self-proposed by various theorists. Goffman (1959) proposed that when people are interacting with others, they strategically present their best self to others, just like actors performing on a stage. Some studies suggested that people highlight positive aspects of themselves or present the ideal self on social network sites in order to create a more socially desirable identity online (e.g., Ranzini and Lutz, 2017; Alsaggaf, 2019).

Self-discrepancy theory was also used frequently as the theoretical background. The theory proposed three domains of the self: the actual self (attributes one current possess), the ideal self (attributes one hopes to possess, reflecting wishes and dreams), and the ought self (attributes one should possess, reflecting duties and responsibilities; Higgins, 1987). People usually use the ideal self and the ought self as self-guides to regulate their behavior. The gap between the actual self and self-guide is referred to as self-discrepancy. Greater self-discrepancy will lead to greater psychological discomfort, such as disappointment and anxiety (Higgins, 1987). The Internet provides opportunities for online identity reconstruction, making it easier to fulfill self-guides and reduce self-discrepancy (Hu et al., 2017). For example, people can create game avatars that are more similar to their ideal self than their actual self (Bessière et al., 2007; Dengah and Snodgrass, 2020). Individuals who are not satisfied with their appearance (i.e., with great actual-ideal self-discrepancy) tend to edit their selfies more frequently (Lyu, 2016).

### Why Do People Reconstruct Their Identity Online?

The Internet gives individuals an avenue to present themselves freely. There is growing evidence that people reconstruct an online identity that is somehow different from their real identity in the offline world (Bessière et al., 2007; Toma and Hancock, 2010; Hu et al., 2015; Jackson and Luchner, 2018). The reasons for online identity reconstruction are complicated.

After reviewing the selected papers, we found that people were mainly driven by various needs during online identity reconstruction. Some people were motivated by social needs. For example, Ranzini and Lutz (2017) found that hooking up/sex and self-validation were important motivations for users of online dating sites to present a deceptive self-image. People want to attract sexual partners and gain self-validation on the dating site (Ranzini and Lutz, 2017). On social network sites, fear of missing out can lead to positive self-presentation (Duan et al., 2020). Hu et al. (2015) suggested that people may reconstruct their online identity due to vanity, enjoyment, access to new social networks, and escape from old social networks (Hu et al., 2015). Other researchers found that adolescents who presented a different identity online were mainly driven by self-exploration, social compensation, and social facilitation (Valkenburg et al., 2005; Ceyhan, 2014). They want to explore the reactions of others, communicate more easily (overcome shyness), and meet new friends (Valkenburg et al., 2005; Ceyhan, 2014). People may also reconstruct an online identity to present their true self (such as traits or beliefs that cannot be easily expressed in the offline world; Hu et al., 2017). In addition to the above-mentioned social needs, people were also motivated by security needs in online identity reconstruction. For instance, people may reconstruct their online identity due to disinhibition, privacy concern, and avoidance of disturbance (Hu et al., 2015). They want to be anonymous and protect themselves online.

Gender and cultural differences were salient in the motivations for online identity reconstruction (Valkenburg et al., 2005; Huang et al., 2020). For example, it is found that when compared with Chinese social network users, Malaysian users were more likely to reconstruct their identity due to privacy concerns (Huang et al., 2020). Girls placed more emphasis on self-exploration and social compensation than boys (Valkenburg et al., 2005), while boys were more likely to be motivated by social facilitation (Ceyhan, 2014). In addition, it is suggested that men are more likely to be motivated by bridging social capital and disinhibition than women (Huang et al., 2018).

### How Do People Reconstruct Their Identity Online?

Positive self-presentation is a strategy used frequently for online identity reconstruction. People try to build a better self-image by presenting themselves positively. Individuals may only share contents that show the good side of their life and avoid posting negative events (Kim and Lee, 2011; Kim and Tussyadiah, 2013; Bareket-Bojmel et al., 2016). Presenting one’s ideal self is also used as a strategy for online identity reconstruction. For example, people may present the “hoped-for possible self” on dating sites to impress others (Yurchisin et al., 2005). On social network sites, individuals often present themselves in the way they want to be (Michikyan et al., 2015; Kang and Wei, 2019). They sometimes use multiple accounts to manage their online identity (Alsaggaf, 2019).

In addition, people may reconstruct their identity by altering the information they post online. For instance, in online dating sites, people often enhance their profile photos (Hancock and Toma, 2009; Toma and Hancock, 2010). Sometimes, they even lie about their physical descriptors (such as height and weight; Toma et al., 2008). It is found that online daters tend to exaggerate their attractiveness by presenting their personality traits in a more desirable way (Guadagno et al., 2012). Online daters build a better identity by deceptive self-presentation in order to improve their attractiveness to potential partners (Ranzini and Lutz, 2017). In addition, individuals also edit the photos they post on social network sites (Fox and Rooney, 2015; Lyu, 2016). People with lower social-economic status try to build a better image online by altering self-presentation, with an attempt to gain social mobility to the upper class (Pitcan et al., 2018). Individuals may also present a false self to deceive others or to explore their identity (Valkenburg and Peter, 2008; Jackson and Luchner, 2018).

### What Factors Will Affect Online Identity Reconstruction Behavior?

In addition to the motivations and strategies for online identity reconstruction, existing studies have identified many other predictors that are significantly associated with the behavior of online identity reconstruction.

#### Personality Traits

Several articles reviewed in this study have examined the role of personality traits in online identity reconstruction (Utz et al., 2012; Fox and Rooney, 2015; Gil-Or et al., 2015; Ranzini and Lutz, 2017). Some personality traits are positively associated with online identity reconstruction. With a sample of 1,000 male social network site users, Fox and Rooney (2015) found that narcissism trait was a significant predictor of photo editing behavior. Highly narcissistic males posted more selfies and edited the photos more frequently to make themselves look better (Fox and Rooney, 2015). Similarly, Utz et al. (2012) indicated that people with a greater need for popularity are more likely to engage in social grooming, profile enhancement, and strategic self-presentation on social network sites (Utz et al., 2012).

While some personality traits promote online identity reconstruction, some traits are negatively associated with this behavior. The effect of self-esteem has attracted much attention from researchers (Gil-Or et al., 2015; Michikyan et al., 2015; Ranzini and Lutz, 2017). Ranzini and Lutz (2017) found that people with a low level of self-esteem tend to present themselves more deceptively in the online dating context. The level of self-esteem can also affect video game players’ construction of game characters (Dengah and Snodgrass, 2020). Using a sample of 258 adult Facebook users, Gil-Or et al. (2015) suggested that self-esteem has negative impacts on false self-presentation, which refers to present oneself in a manner that is inconsistent with who the person really is. Individuals with a higher level of self-esteem are less likely to present a false self on Facebook (Gil-Or et al., 2015). In addition, it is suggested that adolescents with a lower level of self-esteem are more likely to engage in false self-presentation (Michikyan et al., 2015). Sexual orientation can also influence an individual’s behavior of online identity reconstruction (Ranzini and Lutz, 2017). People who identified themselves as homosexual and bisexual were more likely to present themselves in a less authentic way (Ranzini and Lutz, 2017).

#### Physical Attractiveness

Previous research found that people’s concern about their physical appearance had significant influences on online identity reconstruction behavior (Fox and Rooney, 2015; Lyu, 2016). For example, Fox and Rooney (2015) investigated the association between self-objectification (treating one’s appearance as objects that are evaluated by others) and online identity reconstruction with a male sample. Their findings suggested that men with a higher level of self-objectification edit the photos of themselves more frequently on social network sites (Fox and Rooney, 2015). Later, Lyu (2016) found a similar effect of self-objectification on online identity reconstruction with a female sample. Results showed that women who were dissatisfied with their appearance were more likely to engage in photo editing behavior. In addition, women who frequently monitor and compare their appearance with others tend to fabricate their photos purposefully (Lyu, 2016). In addition, Toma and Hancock (2010) found that people with less attractive physical appearances were more likely to enhance their photographs and lie about their height, weight, and age.

#### Psychological Status

Psychological status is another important predictor of online identity reconstruction behavior (Bessière et al., 2007; Valkenburg and Peter, 2008). Bessière et al. (2007) found that online gamers tended to create a game character with attributes that were more favorable than their own attributes, and the discrepancy between game characters and one’s real attributes was greater among players with a lower level of psychological well-being (e.g., a high level of depression; Bessière et al., 2007). This means that players with a lower level of psychological well-being are likely to reconstruct their identity in online games to a greater extent.

Some studies have focused on adolescents’ online identity reconstruction (Valkenburg et al., 2005; Valkenburg and Peter, 2008; Ceyhan, 2014; Michikyan et al., 2015). Adolescents pretend to be someone else online (such as someone older, smarter, less shy, more beautiful) to explore their own identity (Valkenburg et al., 2005). It is suggested that the sense of identity is negatively associated with online identity reconstruction (Ceyhan, 2014; Michikyan et al., 2015). Adolescents with a less coherent sense of the self are more likely to engage in identity experiments on the Internet (Ceyhan, 2014), and present their false self to a greater extent on Facebook (Michikyan et al., 2015). Emerging adults who still have doubts about what they want to be are more likely to engage in online self-exploration to better understand different aspects of themselves (Michikyan et al., 2015). In addition, Valkenburg and Peter (2008) found that loneliness positively predicted online identity experiment. Lonely adolescents explore their identity with online identity reconstruction more frequently than non-lonely peers (Valkenburg and Peter, 2008).

#### Demographic Factors

Demographic variables (such as gender, age, and educational level) can also influence online identity reconstruction. It is found that women’s profile photos were perceived to be less accurate than men’s (Hancock and Toma, 2009), and women indeed engaged in self-enhancement more than men (Toma and Hancock, 2010; Bareket-Bojmel et al., 2016). In addition, age was a negative predictor of false self-presentation, indicating that younger people are more likely to reconstruct their identity online (Wright et al., 2018). Interestingly, Ranzini and Lutz (2017) found that individuals with a higher educational level presented themselves deceptively to a greater extent than those who are less educated.

#### Other Factors

In addition to personality traits, physical attractiveness, psychological status, and demographic variables, existing studies have also examined the role of other factors in online identity reconstruction. For example, Bareket-Bojmel et al. (2016) investigated how the goals of social network site use affect users’ actual behavior on Facebook. Using a sample of 156 undergraduate students, they suggested that students with performance goals (e.g., try to demonstrate their competence to others) have a greater desire for self-enhancement, which in turn, drive them to present themselves in a positive and socially desirable manner to impress others with their competence or talent (Bareket-Bojmel et al., 2016).

Perceived moral norm also has an impact on people’s identity reconstruction behavior on Facebook (Wright et al., 2018). Individuals are more likely to engage in false self-presentation (such as updating status of doing something they did not actually do) if they perceive the behavior of online identity reconstruction is morally acceptable (Wright et al., 2018). For instance, people may lie about their achievements when they think the behavior is acceptable. In the context of online dating, the intention to seek romantic relationships negatively influences deceptive self-presentation (Ranzini and Lutz, 2017). People with long-term relational goals are less likely to engage in self-enhancement (Toma and Hancock, 2010).

### What Are the Effects of Online Identity Reconstruction?

#### Effects on Well-Being

Some studies reported that online identity reconstruction has positive effects on well-being. Kim and Lee (2011) examined the impact of online identity reconstruction on the subjective well-being of Facebook users. Results showed that individuals tend to feel happier when they can present a positive self-image on Facebook, but the positive self-presentation is not likely to improve the perceived social support (Kim and Lee, 2011). Similarly, Jang et al. (2018) found that people who present themselves positively on social network sites reported a higher level of happiness, regardless of their self-esteem level. In addition, it is suggested that selective self-presentation will increase people’s online life satisfaction when they have a low level of self-esteem or have a high level of social trust toward other online users (Kim and Baek, 2014). Moreover, Hu et al. (2020) indicated that online identity reconstruction has a positive impact on psychological well-being. People who reconstruct their identity online tend to feel more autonomous and have a higher level of self-acceptance, which in turn, improves their overall satisfaction online (Hu et al., 2020).

Online identity reconstruction is also associated with negative psychological outcomes (Visser et al., 2013; Wright et al., 2018; Duan et al., 2020). It is suggested that false self-presentation is significantly correlated with negative mental health, such as anxiety, depression, and stress (Wright et al., 2018; Duan et al., 2020). In addition, identity experiments in online games are positively associated with feelings of loneliness. Players who create a game character that is inconsistent with their real identity are likely to feel lonely to a greater extent (Visser et al., 2013).

#### Other Effects

Online identity reconstruction is positively associated with the responses of audiences (Bareket-Bojmel et al., 2016). Individuals who present themselves in a positive and socially desirable way receive more likes and comments from their friends on Facebook (Bareket-Bojmel et al., 2016). It is suggested that trying out ideal selves in MySpace is beneficial for the development of adolescents’ personal identity (Manago et al., 2008). In addition, online identity experiments stimulate adolescents’ communication with people of different ages and from different backgrounds, which in turn, makes them feel a higher level of social competence in the offline world (Valkenburg and Peter, 2008).

#### Moderation and Mediation Effects

In addition to the direct effects, prior studies have also investigated the role of online identity reconstruction as a moderator or mediator. Huang et al. (2019) examined the predictors of online satisfaction. Their findings suggested that bridging social capital and privacy concern have significant impacts on satisfaction, and these influences are moderated by online identity reconstruction. For individuals who reconstruct their identity to a greater extent, the effect of bridging social capital on satisfaction is stronger, while the effect of privacy concern on satisfaction is weaker (Huang et al., 2019). Kim and Tussyadiah (2013) found that social network site use was positively associated with the social support users receive, and this relationship was stronger among users who reconstructed their identity to build a positive self-image. In addition, Jackson and Luchner (2018) found that highly self-critical people (who point out their own perceived flaws frequently) more often presented themselves falsely with a deceptive intention. False self-presentation mediated the relationship between self-criticism and negative emotional response to Instagram feedback (Jackson and Luchner, 2018).

## Discussion

This review has provided an overview of the current trends in online identity reconstruction research. In addition to summarizing the research contexts and sample characteristics, this study revealed that quantitative methods were the preferred methodological approach, and the most popular theories were Goffman’s self-presentation theory and Higgins’ self-discrepancy theory. The analysis of the key findings of existing literature provided insight into the motivations, strategies, predictors, and effects of online identity reconstruction. It is found that people might reconstruct their identity online to fulfill various social needs and security needs, such as to improve sexual attractiveness, explore identity, and protect privacy (Valkenburg et al., 2005; Ceyhan, 2014). The main strategies people use to reconstruct their identity online are positive self-presentation (e.g., presenting their ideal self) and false self-presentation (e.g., altering personal information; Michikyan et al., 2015; Kang and Wei, 2019). Personality traits, physical attractiveness level, psychological status, and demographic factors are important predictors of online identity reconstruction (Bessière et al., 2007; Toma and Hancock, 2010; Utz et al., 2012; Wright et al., 2018). Moreover, online identity reconstruction has influences on individuals’ well-being (such as happiness, perceived support, and depression; Kim and Lee, 2011; Jang et al., 2018; Wright et al., 2018).

Based on the review of relevant articles, we found some weaknesses in online identity reconstruction research in the past years. There are some gaps in existing research. In the following section, we offer critiques and suggestions for possible future directions in online identity reconstruction research.

### Recommendations for Future Research

First, more efforts should be made to explore the effectiveness of online identity reconstruction behavior. Previous research suggested that people may reconstruct their online identity due to different reasons. For example, some people reconstruct their identity to build a positive image and fulfill their need for vanity; some people want to extend their social network (Hu et al., 2015). Although, existing studies have identified various motivations for online identity reconstruction, the effectiveness of online identity reconstruction is not clear. Do people really get what they want (e.g., more positive images and bridging social capital) through online identity reconstruction? Therefore, future studies are suggested to examine the effectiveness of online identity reconstruction. For example, experiments could be designed to evaluate the changes (if any) in perceived physical attractiveness or bridging social capital before and after online identity reconstruction.

Second, the causal relationship between online identity reconstruction and well-being is not clear. As suggested by the reviewed studies, negative psychological status is a significant predictor of online identity reconstruction behavior (Valkenburg et al., 2005; Bessière et al., 2007; Michikyan et al., 2015), while online identity reconstruction is also associated with both positive and negative well-being (Kim and Lee, 2011; Jang et al., 2018; Wright et al., 2018). However, the existing research used cross-sectional data. It is not clear whether well-being affects online identity reconstruction behavior (e.g., promote the behavior), or online identity reconstruction leads to negative (or positive) well-being. Therefore, longitudinal studies are needed to take a closer look at the causal relationship between well-being and online identity reconstruction. Future research can examine the long-term role of well-being in online identity reconstruction.

Third, opportunities exist to investigate the effects of the big five personality traits. Existing studies have examined the effect of personality traits on the behavior of online identity reconstruction (Utz et al., 2012; Fox and Rooney, 2015; Gil-Or et al., 2015). However, they mainly focused on self-esteem or other traits related to physical appearance. There is a lack of research on the effect of the big five personality traits (i.e., Extraversion, Neuroticism, Agreeableness, Conscientiousness, and Openness to Experience). The big five personality traits were found to be significantly associated with self-presentation (Lee et al., 2014; Eşkisu et al., 2017) and motivations for Facebook use (Seidman, 2013). Therefore, it is likely that these personality traits can also influence online identity reconstruction behavior. Thus, future studies can investigate whether the big five personality traits are associated with the motivations for online identity reconstruction or the actual behavior of reconstructing an online identity.

Fourth, there is a lack of research on the potential negative impacts of online identity reconstruction. Some of the reviewed articles have examined online identity reconstruction in the context of online dating (Yurchisin et al., 2005; Ellison et al., 2006; Wotipka and High, 2016; Ranzini and Lutz, 2017). Individuals mispresent their physical characteristics to make themselves more attractive to potential romantic partners (Toma et al., 2008). Given that the online daters are expecting to build a romantic relationship in the offline world (Toma and Hancock, 2010), they only reconstruct their identity to a limited extent. In the context of social network platforms, people may also face some difficulties when they try to reconstruct their identity, because the reconstructed online identity may be judged by their friends who share offline connections with them (Zhao et al., 2008). It is also difficult to control the information posted by others (Rui and Stefanone, 2013). Therefore, it is an interesting direction to investigate whether online identity reconstruction in a non-anonymous environment will induce negative impacts on individuals, such as reduced credibility or negative social evaluations.

### Contributions and Limitations

As a literature review paper that looks closely into the phenomenon of online identity reconstruction, the present study makes several contributions. First, it provides a thorough understanding of online identity reconstruction by identifying the motivations, strategies, predictors, and effects of online identity reconstruction. In addition to these key findings, this study also analyzed the basic characteristics of related research. It provides an overview of the current status of emerging literature. We also identified several knowledge gaps after reviewing existing studies. The recommendations for future research may take the research of online identity reconstruction further. They offer clear directions for researchers in this field. However, this study also has limitations. It should be noted that non-English papers, conference proceedings, and book chapters were excluded from the literature search process. Although, we have increased the coverage of potentially relevant papers by searching several large databases, future reviews are suggested to enlarge the pool of literature selection to achieve a more complete understanding of online identity reconstruction phenomenon.

## Author Contributions

JH, SK, and CH contributed to the conception and design of the study. SK provided the supervision and advice throughout this study. JH and CH contributed to the literature search, literature selection, and data analysis. JH wrote the first draft of the manuscript. All authors contributed to the article and approved the submitted version.

## Conflict of Interest

The authors declare that the research was conducted in the absence of any commercial or financial relationships that could be construed as a potential conflict of interest.

## Publisher’s Note

All claims expressed in this article are solely those of the authors and do not necessarily represent those of their affiliated organizations, or those of the publisher, the editors and the reviewers. Any product that may be evaluated in this article, or claim that may be made by its manufacturer, is not guaranteed or endorsed by the publisher.
